# Dietary Clusters and Mortality Risk in a Chinese Population: The Role of Type 2 Diabetes and Hypertension

**DOI:** 10.3390/nu18111816

**Published:** 2026-06-04

**Authors:** Chuhan Wei, Xikang Fan, Mengxia Li, Zidong Wang, Jiaxi Zhou, Jun Lv, Canqing Yu, Dianjianyi Sun, Pei Pei, Yan Lu, Yujie Hua, Jiang Hua, Jian Su, Jinyi Zhou, Ran Tao

**Affiliations:** 1 School of Public Health, Nanjing Medical University, Nanjing 211166, China; 2 Jiangsu Provincial Center for Disease Control and Prevention, Nanjing 210009, China; 3 Department of Epidemiology & Biostatistics, School of Public Health, Peking University, Xueyuan Road, Haidian District, Beijing 100191, China; 4 Peking University Center for Public Health and Epidemic Preparedness and Response, Beijing 100191, China; 5 Key Laboratory of Epidemiology of Major Diseases (Peking University), Ministry of Education, Beijing 100191, China; 6 Department of Noncommunicable Chronic Disease Control and Prevention, Suzhou Center for Disease Control and Prevention, Suzhou 215004, China; 7 Department of Health Education, Nanjing Municipal Center for Disease Control and Prevention, Nanjing 210003, China

**Keywords:** dietary habits, mortality, type 2 diabetes, hypertension, cluster analysis, prospective cohort

## Abstract

**Background**: Regional dietary variations in China are well-documented, but their mortality associations in local populations, particularly among individuals with type 2 diabetes (T2D) or hypertension, remain unclear. This study aimed to identify dietary clusters in Suzhou and investigate their associations with mortality. **Methods**: This prospective analysis included 53,269 participants aged 30–79 years from the China Kadoorie Biobank (CKB) Suzhou Wuzhong subcohort. The baseline diet was assessed via a food frequency questionnaire, and three dietary clusters were identified by K-means clustering of 10 food groups. Multivariable Cox models were used to estimate hazard ratios (HRs) and 95% confidence intervals (CIs) for all-cause, cardiovascular disease (CVD), diabetes-related, and cancer mortality, stratified by baseline health status, T2D, and hypertension. **Results**: During follow-up, 1263 deaths occurred among healthy adults, 351 in T2D, and 2410 in hypertension. The Traditional/Preserved-Heavy cluster was characterized by lower intake frequencies across most food groups and more frequent preserved vegetable intake; the Meat-Centric cluster by relatively moderate intake frequencies and higher meat and poultry intake; and the Plant-and-Dairy-Abundant cluster by relatively abundant overall dietary intake, more frequent intake of fresh fruit, dairy products, and soybean products, and less frequent preserved vegetable intake. With the Meat-Centric cluster (cluster 2) as the reference, the Plant-and-Dairy-Abundant cluster (cluster 3) in T2D was associated with lower all-cause (HR = 0.61, 95% CI: 0.44–0.85), CVD (0.47, 0.24–0.91), and diabetes-related mortality (0.25, 0.09–0.71). BMI modified the association with all-cause mortality in T2D (*p* interaction = 0.033). In hypertension, cluster 1 was linked to higher all-cause (1.13, 1.03–1.23) and CVD mortality (1.17, 1.00–1.37), whereas cluster 3 was associated with a lower risk of diabetes-related mortality (0.40, 0.16–0.98). **Conclusions**: A dietary habit rich in fruit, dairy products, soybean products, and less frequent preserved vegetable intake was associated with lower mortality risk, particularly in T2D patients, whereas a habit with lower overall intake and more frequent preserved vegetable intake was linked to higher mortality in hypertension participants. These findings should be interpreted in light of the accompanying socioeconomic and lifestyle differences across dietary clusters.

## 1. Introduction

Diet is an important and modifiable lifestyle factor closely related to mortality risk. Recent Global Burden of Disease estimates suggest that poor diet is associated with 12.2% of all deaths in 2023 [1]. Across China, distinct regional dietary habits have emerged, with substantial differences in staple foods and in the consumption of food groups [2,3]. Notably, the dietary habit of the Jiangnan region, where Suzhou is located, has been described as an indigenous Chinese counterpart to the Mediterranean diet and regarded as a dietary habit beneficial to multiple health outcomes [4]. But even within Suzhou, dietary habits still varied across individuals, and the specific dietary habits in the local population and their potential health effects have not been well characterized.

Suzhou Wuzhong District, where our present study was conducted, is located in southern Suzhou along Taihu Lake and represents a mixed urbanizing setting within the Jiangnan region. Although it is an important part of Suzhou’s main urban area, Wuzhong also retains lakeside towns, traditional villages, agricultural resources, and water-town cultural features. Local livelihoods span agriculture, fisheries, small businesses, manufacturing, and service-sector work, while daily mobility is supported by road, public transport, and waterway networks connecting lakeside settlements with the central urban area. Culturally, the area is embedded in Wu/Jiangnan foodways, characterized by rice-based staples, freshwater products, tea, seasonal fruits, and traditional preserved foods. Therefore, identifying locally relevant dietary clusters and examining their associations with mortality may help clarify how everyday dietary habits are related to long-term health risks in this urbanizing Jiangnan population.

Variation in dietary habits may be particularly relevant in high-risk populations, such as individuals with type 2 diabetes (T2D) and hypertension. These two conditions are closely interrelated cardiometabolic disorders that frequently coexist and jointly contribute to increased risks of cardiovascular and renal complications, substantial disease burden, premature death, and reduced life expectancy [5,6]. Existing evidence suggests that adherence to healthy dietary habits may be beneficial for prognosis in these high-risk populations. Among individuals with T2D, higher intakes of fish, whole grains, fiber, n-3 polyunsaturated fatty acids, and greater adherence to Mediterranean-style diets, have been associated with lower all-cause and cardiovascular disease (CVD) mortality [7,8]. Similar associations have also been reported in hypertension, where higher adherence to healthy dietary habits, including the Mediterranean diet, Alternative Healthy Eating Index (AHEI), and Dietary Approaches to Stop Hypertension (DASH), has also been associated with lower all-cause and CVD mortality [9,10].

However, most prospective studies on dietary and mortality have focused on either general populations or a single disease group, and evidence comparing such associations across populations with different baseline health status within the same dietary background remains limited. Moreover, dietary scores such as the Dietary Inflammatory Index, HEI, and Mediterranean diet scores primarily reflect adherence to predefined dietary patterns, whereas cluster analysis is a data-driven approach that can identify locally relevant combinations of food groups based on individual food intake characteristics and classify local residents with similar dietary behaviors [11,12].

Therefore, using data from a prospective cohort in Suzhou, we identified three dietary clusters and examined their associations with all-cause and cause-specific mortality in participants who were relatively healthy at baseline and those with T2D and hypertension. Additionally, we further explored whether these associations varied by subgroups.

## 2. Materials and Methods

### 2.1. Study Population

The China Kadoorie Biobank (CKB) is a large-scale prospective cohort with 512,723 participants from 10 different geographical regions in China who were aged 30–79 years at enrollment. The current study used data from the Suzhou Wuzhong subcohort of the CKB, which is one of the 5 urban regions in the CKB cohort. The baseline survey was conducted between 2004 and 2008, and 53,269 participants were enrolled to complete baseline information. Further details of the CKB study design have been described elsewhere [13,14,15]. This study was conducted in accordance with the Declaration of Helsinki. Ethical approval was obtained from the Ethical Review Committee of the Chinese Center for Disease Control and Prevention (protocol code 005/2004, date of approval: 11 March 2004, Beijing, China) and the Oxford Tropical Research Ethics Committee, University of Oxford (protocol code 025-04, date of approval: 2 December 2004). All participants provided written informed consent.

All participants completed a laptop-based electronic questionnaire administered face-to-face by trained interviewers at baseline and underwent standardized physical measurements. The baseline questionnaire collected information on demographic characteristics, lifestyle, clinical conditions, and family history. Physical measurements included height, weight, and blood pressure.

According to baseline disease status, we divided the cohort into three analytical populations: a baseline healthy population, patients with T2D, and patients with hypertension. The baseline healthy population was defined as participants without coronary heart disease, stroke or transient ischemic attack, cancer, emphysema and chronic bronchitis, T2D, or hypertension at baseline. Participants were classified as having baseline T2D if they had a fasting blood glucose of ≥7.0 mmol/L, random blood glucose of ≥11.1 mmol/L, self-reported or physician-diagnosed diabetes, or current use of any glucose-lowering medications at baseline, with individuals with type 1 diabetes (ICD-10: E10) excluded [16]. Patients with hypertension were defined as those with a systolic blood pressure of ≥140 mmHg, diastolic blood pressure of ≥90 mmHg, self-reported or physician-diagnosed hypertension, or current use of antihypertensive medications [17].

### 2.2. Assessment of Dietary Clusters

Dietary information at baseline was collected using a food frequency questionnaire (FFQ). Face-to-face interviews were conducted to obtain information on the frequency of intake of 12 major food groups over the past year, with response options including daily, 4–6 days per week, 1–3 days per week, monthly, and never or rarely. The questionnaire included 12 food groups: rice, wheat-based foods, coarse grains, meat, poultry, fish, eggs, fresh vegetables, soybean products, preserved vegetables, fresh fruits, and dairy products. Among these, the intake frequency of rice and fresh vegetables showed little variability, reported as daily in more than 99% of participants. Therefore, these variables were not included in subsequent analyses [18]. The qualitative FFQ was repeated in a subsample of 926 participants within one year after baseline and showed good reproducibility of the food and beverage items, and a subsequent validation study against repeated 24 h dietary recalls further supported its relative validity [19].

### 2.3. Cluster Analysis

Before performing cluster analysis, a near-zero variance test was conducted on the 10 dietary variables to avoid the influence of variables with extremely low variability on the clustering results, and no variables were identified [20]. We then converted the original frequency categories into ordered numerical scores ranging from 0 to 4, where 0 indicated never or rarely, 1 indicated a few times per month, 2 indicated 1–3 days per week, 3 indicated 4–6 days per week, and 4 indicated daily intake. All dietary variables were subsequently standardized using Z-scores to eliminate the impact of differences in measurement scales on the clustering results.

Dietary clusters were identified using the K-means clustering method. The clustering analysis was performed based on standardized dietary variables, with the objective of minimizing the within-cluster sum of squares (WSS) [21]. For each candidate clustering solution, the algorithm was run 25 times with different random initial centroids, and the solution with the smallest total WSS was retained. A random seed was set to ensure reproducibility. To determine the optimal number of clusters, we evaluated *k* values from 1 to 10 using the elbow method and plotted the corresponding elbow curve. When *k* increased from 1 to 3, the reduction in WSS was substantial. Beyond *k* = 3, the decrease in WSS became more gradual, indicating limited improvement in model fit with additional clusters (Supplementary Figure S1). The three-cluster solution was nutritionally meaningful and relatively simple. It separated participants into three distinct and locally meaningful dietary clusters, which captured the major dietary contrasts in this population while avoiding excessive subdivision of dietary behaviors, and included sufficient numbers of participants for subsequent mortality analyses. The alternative *k* = 2 and *k* = 4 solutions were also explored for comparison (Supplementary Figures S2 and S3). Thus, the three-cluster solution was selected because it provided a balance between statistical performance, clustering stability, simplicity, adequate sample size within clusters, and clinically/nutritionally meaningful interpretation. All participants were accordingly classified into three dietary clusters.

To assess the stability of the three-cluster results, we repeated K-means clustering with different random seeds with *k* fixed at 3, and the Adjusted Rand Index (ARI) was used for evaluation [22]. The ARI values ranged from 0.997 to 1.000, indicating high stability of the clustering results and supporting their suitability for subsequent analyses (Supplementary Figure S4).

### 2.4. Assessment of Covariates

Demographic characteristics comprised age, sex, highest education level (primary school or below, middle school/technical secondary school, college or above), household income (<20,000, 20,000–34,999, ≥35,000 Chinese yuan per year), and marital status (married, unmarried, widowed/separated/divorced). Lifestyle-related variables included smoking (non-smoker; occasional smoker; ever-smoker; current smoker) [23], alcohol consumption (non-drinker; ever-drinker; occasional drinker; current drinker) [24], and physical activity level. Daily physical activity was quantified as metabolic equivalent of task hours per day (MET-h/d), calculated as the sum of MET values multiplied by the duration (hours/day) for all reported activities. Clinical measurements included body mass index (BMI), calculated as weight (kg) divided by height squared (m^2^), and blood pressure (mmHg). In addition, clinical conditions included baseline hypertension status (yes or no), T2D status (yes or no), and duration of diabetes (years). Family history variables included stroke, myocardial infarction, diabetes, and cancer (yes or no). For women, menopausal status was categorized as not yet menopausal, currently undergoing menopause, or post-menopausal.

### 2.5. Ascertainment of Mortality

Follow-up for the present study started from the completion of the baseline survey and continued until death, loss to follow-up, or 31 December 2018, whichever occurred first. Mortality data were obtained from local death registries, residential records, and the national health insurance system. For individuals who were lost to follow-up, the date of loss to follow-up was treated as the censoring date. The cause of death was coded by trained staff, blinded to baseline information of participants, using the International Classification of Diseases, 10th Revision (ICD-10). In the present study, only the underlying cause of death was included in the analysis. The outcomes comprised death from all-cause mortality, as well as selected disease-specific causes, including CVD mortality (ICD-10: I00-I99), diabetes-related mortality (ICD-10: E10-E14), and cancer mortality (ICD-10: C00-C97).

### 2.6. Statistical Analyses

The three dietary clusters identified by cluster analysis were the main exposure groups. Baseline continuous variables were presented as means ± standard deviations (SDs), and differences across dietary clusters were compared using a one-way analysis of variance. Categorical variables were presented as numbers and percentages, and differences across dietary clusters were compared using the chi-square test. Multivariable Cox proportional-hazard regression models were used to estimate the associations of different dietary clusters with the risks of all-cause and cause-specific mortality, with hazard ratios (HRs) and 95% confidence intervals (CIs) calculated. The second cluster was used as the reference group and two multivariable models were constructed: (1) In all three populations, Model 1 was adjusted for age and sex only. (2) In the baseline healthy population, Model 2 was further adjusted for highest education level, annual household income, marital status, physical activity, smoking, alcohol consumption, BMI, and menopausal status in women, with additional adjustment for corresponding variables according to different causes of death, including family history of stroke, myocardial infarction, diabetes, and cancer for all-cause mortality; family history of stroke and myocardial infarction for CVD mortality; family history of diabetes for diabetes-related mortality; and family history of cancer for cancer mortality. In participants with T2D and hypertension, Model 2 was based on the fully adjusted model for the baseline healthy population and further extended the fully adjusted model for the baseline healthy population with additional covariates: duration of diabetes and baseline hypertension status for T2D patients, and baseline diabetes status and systolic blood pressure for hypertensive patients. The proportional-hazard assumption was tested using Schoenfeld residuals and no substantial violation was observed.

Subgroup analyses were also performed to evaluate the heterogeneity in the associations among different dietary clusters and the risks of all-cause and cause-specific mortality, stratified by sex, age, BMI, smoking, and alcohol consumption.

In addition, a sensitivity analysis, in which participants who died during the first year of follow-up were excluded, was performed to assess the reliability of the association results. All statistical analyses were performed using R Statistical Software (version 4.4.1; R Foundation for Statistical Computing, Vienna, Austria); two-sided *p* values < 0.05 were considered statistically significant.

## 3. Results

### 3.1. Cluster Characteristics

**Figure 1:**
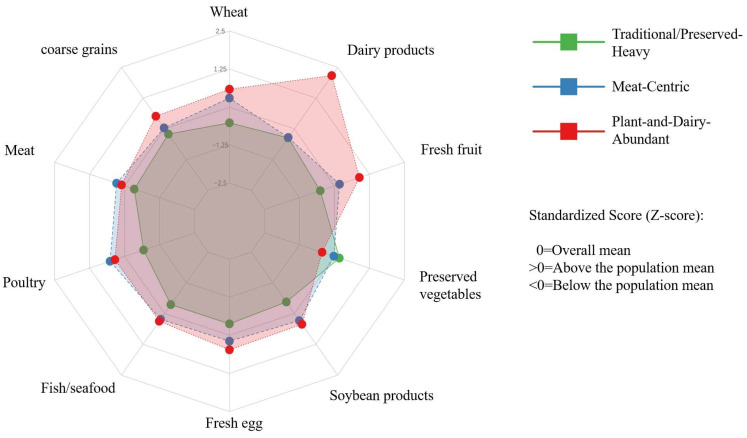
Radar plot of standardized dietary profiles across the three dietary clusters. The plot shows the mean standardized scores (Z-scores) of the 10 food groups included in the cluster analysis for each dietary cluster. Dietary clusters were identified using K-means clustering based on standardized dietary variables. Higher values indicate intake frequencies above the population mean, whereas lower values indicate intake frequencies below the population mean. Cluster 1 (Traditional/Preserved-Heavy) was characterized by a lower overall intake and higher preserved vegetable intake; cluster 2 (Meat-Centric) by higher meat, poultry, and aquatic product intake; and cluster 3 (Plant-and-Dairy-Abundant) by a higher intake of wheat, coarse grains, eggs, soybean products, fresh fruit, and dairy products, with the lowest preserved vegetable intake.

Three distinct clusters were identified in this population and were labelled according to their dominant dietary characteristics: the Traditional/Preserved-Heavy cluster (cluster 1), the Meat-Centric cluster (cluster 2), and the Plant-and-Dairy-Abundant cluster (cluster 3). The Kruskal–Wallis test revealed that food groups were consumed at different levels across the three clusters. Overall, 21,970 (41.2%) participants were classified into the Traditional/Preserved-Heavy cluster, 23,751 (44.6%) into the Meat-Centric cluster, and 7548 (14.2%) into the Plant-and-Dairy-Abundant cluster. The Traditional/Preserved-Heavy cluster was characterized by lower intake frequencies across most food groups and more frequent preserved vegetable intake; the Meat-Centric cluster by relatively moderate intake frequencies and higher meat and poultry intake; and the Plant-and-Dairy-Abundant cluster by relatively abundant overall dietary intake, more frequent intake of fresh fruit, dairy products, and soybean products, and the lowest preserved vegetable intake (Table 1 and Figure 1).

### 3.2. Baseline Characteristics of Participants

A total of 53,269 participants were included in the present analysis, with a mean ± SD age of 52.10 ± 10.35 years at baseline. A total of 58.0% were women (Table 2). 92.7% of participants were married, 60.8% were non-smokers, and 58.8% were non-drinkers. The overall BMI and systolic blood pressure were 24.03 ± 3.21 kg/m^2^ and 132.76 ± 20.32 mmHg, respectively. In addition, 4.6% of participants had baseline diabetes and 39.7% had baseline hypertension.

Baseline characteristics differed significantly across the three dietary clusters for nearly all variables (all *p* < 0.05), except family history of cancer (*p* = 0.755). Traditional/Preserved-Heavy participants tended to be older, more often women, and had lower educational attainment and household income and higher systolic blood pressure. The Meat-Centric cluster featured a higher proportion of males, current smokers, current drinkers, and had higher levels of physical activity and BMI. The Plant-and-Dairy-Abundant cluster was characterized by higher educational attainment and household income, lower systolic blood pressure and levels of physical activity, and a higher prevalence of baseline diabetes and family history of cardiometabolic diseases. Family history of cancer was broadly similar across the three clusters. Baseline characteristics of the three clusters within the healthy, T2D, and hypertension populations are presented in Supplementary Tables S1–S3, respectively.

### 3.3. Association of Dietary Clusters and Mortality

During a median follow-up of 11.12 years, 1263 all-cause deaths occurred among healthy adults, 351 among patients with T2D, and 2410 among patients with hypertension. Detailed numbers of events and mortality rates for all-cause and cause-specific mortality are shown in Table 3.

In the fully adjusted model, no significant associations were observed of dietary clusters with all-cause, CVD, diabetes-related, or cancer mortality among healthy adults. Among patients with T2D, the Plant-and-Dairy-Abundant cluster was associated with lower risks of all-cause, CVD, and diabetes-related mortality than the Meat-Centric cluster, with adjusted HRs of 0.61 (95% CI: 0.44–0.85), 0.47 (95% CI: 0.24–0.91), and 0.25 (95% CI: 0.09–0.71), respectively. For diabetes-related mortality, 59 deaths occurred among 2442 T2D participants, with a mortality rate of 2.13 per 1000 person-years. No significant association was observed for cancer mortality. Among patients with hypertension, the Traditional/Preserved-Heavy cluster was associated with a modestly higher risk of all-cause mortality (HR = 1.13, 95% CI: 1.03–1.23) and, to a lesser extent, CVD mortality (HR = 1.17, 95% CI: 1.00–1.37), while the Plant-and-Dairy-Abundant cluster was associated with a lower risk of diabetes-related mortality (HR = 0.40, 95% CI: 0.16–0.98). This endpoint included 66 deaths among 21,129 participants, with a mortality rate of 0.27 per 1000 person-years.

### 3.4. Subgroup and Sensitivity Analyses

**Figure 2:**
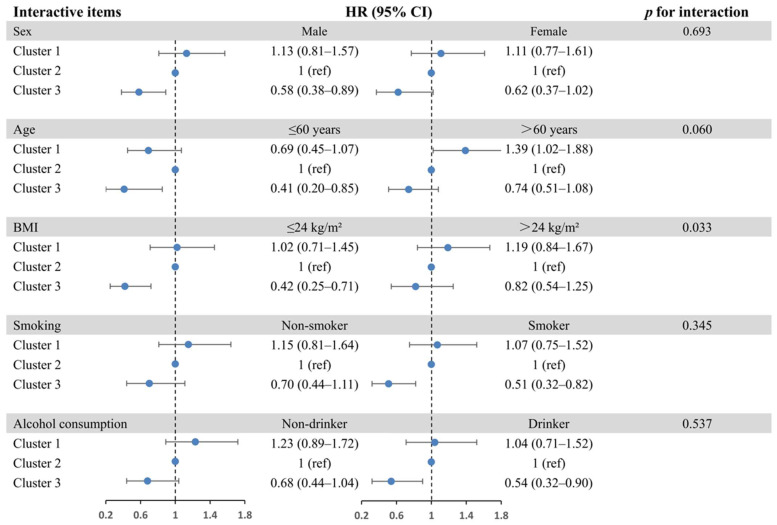
Subgroup analysis of the associations between dietary clusters and all-cause mortality risk among patients with type 2 diabetes. Cluster 1: Traditional/Preserved-Heavy cluster; cluster 2: Meat-Centric cluster; cluster 3: Plant-and-Dairy-Abundant cluster. The HRs (95% CIs) for all-cause mortality were estimated using Cox proportional-hazard models, with cluster 2 as the reference group. Analyses were stratified by sex, age, body mass index (BMI), smoking status, and alcohol consumption. The fully adjusted model included age, sex, highest education level, annual household income, marital status, physical activity, smoking, alcohol consumption, BMI, menopausal status in women, and family history of relevant diseases; for patients with type 2 diabetes, duration of diabetes and baseline hypertension status were additionally adjusted for. BMI: body mass index; CIs: confidence intervals; HRs: hazard ratios.

In subgroup analyses stratified by sex, age, BMI, smoking, and alcohol consumption, no significant heterogeneity was observed in the associations between dietary clusters and all-cause mortality among healthy adults or patients with hypertension (all *p* for interaction > 0.05; Supplementary Figures S5 and S6). Among patients with T2D, however, BMI significantly modified the association (*p* for interaction = 0.033). Compared with the Meat-Centric cluster, the Plant-and-Dairy-Abundant cluster was associated with a lower risk of all-cause mortality among participants with a BMI ≤ 24 kg/m^2^ (HR = 0.42, 95% CI: 0.25–0.71), whereas the association was not statistically significant among those with a BMI > 24 kg/m^2^. No significant interactions were found for sex, age, smoking, or alcohol consumption (Figure 2).

In sensitivity analyses, the associations of dietary clusters with the risk of each event did not materially change after exclusion of deaths occurring within the first year of follow-up (Supplementary Table S4). These findings suggest that the main results were generally robust.

## 4. Discussion

In this community-based prospective cohort from Wuzhong, we identified three distinct dietary clusters and found that their associations with mortality differed by baseline disease status. The Plant-and-Dairy-Abundant cluster (cluster 3) was associated with lower risks of all-cause, CVD, and diabetes-related mortality among participants with T2D, and with a lower risk of diabetes-related mortality among those with hypertension, whereas the Traditional/Preserved-Heavy cluster (cluster 1) was associated with a modestly higher risk of all-cause mortality among participants with hypertension. However, findings of diabetes-related mortality are hypothesis-generating, as they rely on relatively few events and imprecise estimates. BMI also significantly modified the association in T2D.

Nationwide studies of Chinese dietary habits and transitions identified three distinct adult patterns: southern, modern, and meat. From 1991 to 2015, southern pattern scores declined while modern/meat pattern scores rose, indicating gradual dietary westernization in China [25]. In contrast, 41.2% of participants in Suzhou, a representative Jiangnan city, still followed a traditional low-intake, high-preserved-vegetable diet (>4–6 days/week). This dietary cluster reflects local customs, climate, culture and socioeconomic conditions, and aligns with our study’s baseline characteristics. Additionally, 14.2% of participants exhibited a diet similar to the recently proposed Jiangnan diet, marked by a high intake of seasonal vegetables and fruits, freshwater fish, shrimp and soy products; a moderate intake of whole-grain rice and red meat; and a low intake of salt [4]. This cluster conforms to current dietary guidelines and may represent a potentially favorable dietary habit in southern Chinese and broader East Asian populations [4].

Our three-cluster model partially aligned with prior dietary pattern analyses, with distinct local features. A study in older Chinese adults identified healthy, Western and balanced patterns: their healthy pattern matched our Plant-and-Dairy-Abundant cluster, as both were characterized by a high intake of nutrient-dense foods such as fruits, fish, and soybean products, while their Western pattern resembled our Meat-Centric cluster, though our local cluster lacked typical Western fast food, sweets and processed foods [26]. Another CKB study identified traditional southern, traditional northern and Western/new affluence patterns: the Western/new affluence dietary pattern resembled our Plant-and-Dairy-Abundant cluster in showing higher intake of fresh fruit, fish, eggs, and dairy products, whereas the traditional northern dietary pattern, although primarily reflecting the classification of staple foods such as rice or wheat, was similar to our Traditional/Preserved-Heavy cluster in also representing a relatively simple dietary habit with fewer protective foods [27].

A critical caveat is that the dietary clusters identified in this study should not be interpreted as pure indicators of diet alone. Although they were derived from food-frequency variables, the clusters were accompanied by distinct socioeconomic, clinical, and lifestyle profiles, including differences in education, household income, smoking, alcohol consumption, physical activity, blood pressure, and baseline disease status [28,29]. For example, the Meat-Centric cluster included higher proportions of current smokers and drinkers and higher total physical activity, whereas the Plant-and-Dairy-Abundant cluster had a more nutrient-dense dietary habit but lower total physical activity and more advantaged socioeconomic characteristics. Although we adjusted for a wide range of measured demographic, socioeconomic, lifestyle, clinical, and family-history variables and excluded first-year deaths in sensitivity analyses, residual confounding may remain because some relevant factors, including occupational conditions, physical activity domains, health-care access, medication adherence, disease management, and diet-related health consciousness, were not collected or only partially captured. Therefore, the observed associations should be interpreted as associations of dietary clusters embedded within broader socioeconomic and lifestyle contexts, rather than as isolated causal effects of diet alone.

Of the dietary clusters identified in this study, the Plant-and-Dairy-Abundant cluster (cluster 3) was characterized by more frequent intakes of fresh fruit, dairy products, soybean products, and coarse grains, together with the lowest intake of preserved vegetables. A previous systematic review and dose-response meta-analysis demonstrated that fruit consumption was associated with an 18% lower risk of all-cause mortality and a 10% lower risk of CVD mortality [30], while in the CKB, among 26,139 adults with T2D, consuming soy foods at least 4 days per week was associated with lower CVD mortality than never consuming (HR = 0.77, 95% CI: 0.62–0.96) [31]. Long-term high dietary fiber intake has been associated with lower mortality risk in patients with T2D, as evidenced by the US Nurses’ Health Study which reported that a higher intake of these foods was associated with lower all-cause (RR = 0.72, 95% CI: 0.56–0.92) and CVD mortality (RR = 0.65, 95% CI: 0.43–0.99) [32]. A similar inverse association was also observed for the Plant-and-Dairy-Abundant cluster in our study, particularly among participants with T2D.

T2D patients generally exhibit more severe metabolic disturbance and systemic inflammation than relatively healthy adults, leading to higher risks of all-cause and CVD mortality. Healthy dietary habits may be associated with better prognosis in T2D, partially mediated by inflammation. Compared with a Meat-Centric diet, a Plant-and-Dairy-Abundant diet may provide more high-quality protein, minerals, and antioxidant bioactive compounds, which could be relevant to cardiometabolic pathways involving inflammation and oxidative stress. Recent mechanistic reviews suggest that dietary fiber, polyphenols, antioxidant vitamins, and omega-3 fatty acids regulate inflammation and oxidative balance. Quercetin, a common plant/fruit flavonol, has antioxidant, anti-inflammatory, antidiabetic, and cardioprotective effects. These mechanisms support the inverse association between the fruit-rich Plant-and-Dairy-Abundant cluster and mortality, especially in T2D participants [33,34]. In vitro studies and meta-analyses demonstrate that fruits and whole grains exert anti-inflammatory effects by modulating inflammatory cell signaling through antioxidants and polyphenols, and suppressing the production of C-reactive protein (CRP), tumor necrosis factor-α (TNF-α) and interleukin-6 (IL-6) via inhibiting gene expression [35,36,37]. Fresh fruit and soy products have also been reported to improve antioxidant capacity and reduce lipid peroxidation and oxidative DNA damage [38,39]. By contrast, dietary habits characterized by a higher intake of red and processed meat may increase inflammatory and oxidative burden through pro-inflammatory compounds generated during cooking and processing [40]. Thus, these findings provide biological plausibility for the inverse associations observed for the Plant-and-Dairy-Abundant cluster among participants with T2D. Nevertheless, residual socioeconomic and lifestyle confounding remains possible, and the observed inverse association may reflect both dietary composition and correlated lifestyle or socioeconomic factors. Repeated dietary assessments and more detailed information on physical activity domains, smoking and drinking intensity, occupational conditions, health-care access, medication adherence, and socioeconomic trajectories would help clarify the independent contribution of diet.

The less favorable association observed for the Traditional/Preserved-Heavy cluster (cluster 1) among participants with hypertension in all-cause and CVD mortality may be related to its characteristic combination of lower overall dietary diversity and more frequent preserved vegetable intake. Global dietary guidelines recommend increasing dietary diversity to ensure sufficient essential nutrients and optimal health [41]. Higher dietary diversity is linked to lower exposure to toxic ingredients in food and optimized telomere length [42], while insufficient diversity may result in inadequate overall nutrient adequacy, as well as insufficient intake of high-quality protein, dietary fiber, potassium, calcium and various antioxidant compounds, thereby impairing the blood-pressure-lowering and cardiovascular protective effects of diet [43,44]. This interpretation is broadly consistent with previous evidence in the hypertensive population. In a US National Health and Nutrition Examination Survey (NHANES) analysis of 6690 hypertension adults, higher HEI-2010 scores, which indicate better nutritional adequacy, were associated with a 32% reduction in all-cause mortality (HR = 0.68, 95% CI: 0.54–0.85) [45].

Additionally, Chinese evidence further supports the interpretation of the preserved vegetable component of the Traditional/Preserved-Heavy diet. In the CKB, preserved vegetable consumption was associated with marginally higher CVD mortality, with HRs of 1.07–1.12 across increasing intake categories [46], while the Chinese Longitudinal Healthy Longevity Survey similarly reported that all-cause mortality was 10% higher among the oldest old who consumed salt-preserved vegetable [47]. Preserved vegetables in the Suzhou area, unlike the fermented sour pickles commonly consumed in northwestern China, are predominantly non-fermented salt-preserved vegetables [48], whose higher sodium burden may be more detrimental to hypertension patients. Experimental and multi-omics evidence has shown that hypertensive patients are substantially more salt-sensitive than normotensive individuals: the prevalence of salt sensitivity is about 26% in normotensive adults but reaches 51% in patients with hypertension, representing an approximately 1.96-fold difference [49,50]. High sodium intake is an important environmental risk factor for hypertension and may promote disease progression by increasing volume load and vascular calcification, thereby elevating the risks of CVD and mortality [51]. Moreover, during vegetable preservation, microbial degradation may convert nitrate to nitrite, leading to contamination with N-nitroso compounds, which may cause cumulative injury and induce endothelial dysfunction [46]. Therefore, patients with hypertension may be particularly susceptible to the Traditional/Preserved-Heavy diet. Among high-risk individuals with pre-existing hypertension, this dietary cluster was associated with higher mortality risk. However, the less favorable association observed for the Traditional/Preserved-Heavy cluster should not be attributed solely to preserved vegetable intake or sodium burden. The socioeconomic and clinical profile of this cluster suggests that this cluster may also reflect socioeconomic disadvantage, constrained food choices, persistence of traditional dietary habits, and potentially less optimal hypertension management [52]. Although exclusion of first-year deaths reduced potential reverse causality, baseline disease severity, health status, or post-diagnosis dietary changes may still have influenced dietary choices. A more detailed assessment of sodium intake, preserved vegetable consumption and socioeconomic conditions would help clarify the relative contributions of dietary and non-dietary factors.

We also found that BMI modified the association between dietary clusters and all-cause mortality among T2D participants, with the inverse association of the Plant-and-Dairy-Abundant cluster more pronounced in those with a BMI ≤ 24 kg/m^2^. This aligns with prior findings showing stronger associations between high glycemic load/carbohydrate/sugar intake and mortality in leaner (BMI ≤ 25 kg/m^2^) diabetic individuals [53]. A possible explanation is that participants with a lower BMI may have a lower burden of obesity-related metabolic abnormalities, making the association between dietary composition and mortality more detectable. In contrast, among participants with higher BMI, obesity-related insulin resistance, chronic low-grade inflammation, blood pressure elevation, dyslipidemia, and other cardiometabolic risks may outweigh or obscure the potential association between dietary composition and mortality [54]. However, this subgroup finding should be interpreted cautiously because BMI is an imperfect proxy for adiposity and metabolic health, and the result may also reflect limited events, residual confounding, or unmeasured disease severity.

The evidence for cause-specific mortality, particularly diabetes-related mortality, was limited by small event counts. Diabetes-related deaths were few across analytical populations, and some subgroup analyses had limited events, resulting in wide confidence intervals, low statistical power, and potentially unstable effect estimates. Thus, these results are exploratory rather than definitive and require confirmation in larger cohorts or pooled analyses with sufficient cause-specific events.

Our study has several strengths, including a large sample size, adjustment for multiple confounders, long and nearly complete follow-up, and the availability of a wide range of causes of death. In addition, we assessed associations separately in relatively healthy adults, participants with T2D, and participants with hypertension, allowing us to examine potential heterogeneity by baseline disease status. However, it also has limitations. First, dietary information was collected using a qualitative FFQ, and only intake frequency rather than portion size, absolute intake, total energy intake, or nutrient intake amount was available. Therefore, we were unable to quantify total energy intake, sodium intake, or the actual amounts of preserved vegetables consumed. This limitation is critical for interpreting the association between the Traditional/Preserved-Heavy cluster and mortality among participants with hypertension, as we cannot determine whether the observed association is attributable to sodium burden, preservation-related compounds, preparation methods, or broader dietary and socioeconomic characteristics. Second, dietary habits were assessed at baseline, precluding the full capture of follow-up changes. Over the decade, socioeconomic development may have improved overall diets, as shown by reduced preserved vegetable intake in our cohort [48]. This non-differential misclassification may have attenuated the observed associations [55]. Third, the cause-specific mortality analyses, particularly those for diabetes-related mortality, were based on relatively small numbers of events in some analytical populations and subgroups. These findings should therefore be considered exploratory rather than definitive. Fourth, detailed baseline lipid parameters (LDL-C, HDL-C, triglycerides, total cholesterol, non-HDL-C, ApoB, Lp(a)) were unavailable in this analysis. We therefore could not characterize lipid profiles across dietary clusters or perform additional adjustments or sensitivity analyses for these markers. As dietary clusters may correlate with lipid metabolism, lipid-lowering treatment, and residual cardiovascular risk, unmeasured lipid-related confounding remains possible. Fifth, dietary clusters derived from the overall population may not fully capture the within-group dietary heterogeneity of specific disease-defined subgroups. Finally, residual confounding remains an important limitation. Although we adjusted for a range of demographic, lifestyle, and clinical factors, these variables may not fully capture socioeconomic position, occupational conditions, smoking and drinking intensity, physical activity domains, health-care access, diet-related health consciousness, medication adherence, or long-term disease management. Therefore, the observed associations should be interpreted as associations of dietary clusters embedded within broader lifestyle and socioeconomic contexts, rather than as isolated effects of diet alone.

## 5. Conclusions

The Plant-and-Dairy-Abundant cluster, characterized by more frequent intake of several nutrient-dense food groups and less frequent preserved vegetable intake, was linked to lower mortality risk, particularly among participants with T2D, whereas the Traditional/Preserved-Heavy cluster, characterized by lower overall intake and more frequent preserved vegetable intake, was associated with higher mortality in those with hypertension. These findings may provide useful evidence for dietary recommendations targeting local high-risk populations.

## Figures and Tables

**Table 1:** Mean raw dietary frequency scores of the 10 food groups across the three dietary clusters (Mean ± SD).

Food Groups	Traditional/Preserved-Heavy		Meat-Centric		Plant-and-Dairy-Abundant	
	Scores	Rank	Scores	Rank	Scores	Rank
Wheat	0.81 (0.80)	3	1.66 (0.98)	2	1.96 (1.07)	1
Coarse grains	0.50 (0.52)	3	0.65 (0.55)	2	0.92 (0.75)	1
Meat	2.28 (0.91)	3	2.90 (0.94)	1	2.72 (0.97)	2
Poultry	0.80 (0.64)	3	1.69 (0.57)	1	1.56 (0.67)	2
Fish/sea food	1.77 (0.74)	3	2.21 (0.67)	2	2.27 (0.72)	1
Fresh eggs	1.63 (0.79)	3	2.12 (0.75)	2	2.36 (0.97)	1
Soybean products	1.43 (0.70)	3	2.00 (0.66)	2	2.11 (0.81)	1
Preserved vegetables	3.18 (1.20)	1	2.93 (1.29)	2	2.38 (1.41)	3
Fresh fruit	1.47 (0.88)	3	2.27 (1.10)	2	3.08 (1.06)	1
Dairy products (milk, yogurt)	0.14 (0.49)	3	0.15 (0.43)	2	3.15 (1.00)	1

Notes: Cluster 1: Traditional/Preserved-Heavy cluster; cluster 2: Meat-Centric cluster; cluster 3: Plant-and-Dairy-Abundant cluster. Scores: Mean FFQ-derived dietary frequency scores for each food group within each cluster. Three dietary clusters were identified by K-means cluster analysis; food group ranks were obtained from cross-cluster comparisons of intake scores. All *p* values for between-cluster differences were <0.0001.

**Table 2:** Characteristics of participants in the subcohort of the China Kadoorie Biobank study in Suzhou.

Characteristics	Overall	Traditional/Preserved-Heavy	Meat-Centric	Plant-and-Dairy-Abundant	*p*
No. of participants	53,269	21,970	23,751	7548	
Age, y	52.10 (10.35)	53.60 (10.26)	51.03 (9.98)	51.09 (11.18)	<0.001
Women	30,903 (58.01%)	14,543 (66.19%)	11,670 (49.13%)	4690 (62.14%)	
Highest education level					
Primary school or below	15,934 (29.91%)	9519 (43.33%)	5541 (23.33%)	874 (11.58%)	<0.001
Middle/technical secondary school	32,131 (60.32%)	11,699 (53.25%)	15,813 (66.58%)	4619 (61.20%)	
College or above	5204 (9.77%)	752 (3.42%)	2397 (10.09%)	2055 (27.23%)	
Household income					
<20,000 RMB/year	14,196 (26.65%)	8388 (38.18%)	4765 (20.06%)	1043 (13.82%)	<0.001
20,000~34,999 RMB/year	16,809 (31.55%)	7529 (34.27%)	7303 (30.75%)	1977 (26.19%)	
≥35,000 RMB/year	22,264 (41.80%)	6053 (27.55%)	11,683 (49.19%)	4528 (59.99%)	
Marital status					
Married	49,395 (92.73%)	19,853 (90.36%)	22,501 (94.74%)	7041 (93.28%)	<0.001
Widowed/separated/divorced	3717 (6.98%)	2021 (9.20%)	1205 (5.07%)	491 (6.51%)	
Unmarried	157 (0.29%)	96 (0.44%)	45 (0.19%)	16 (0.21%)	
Physical activity level, MET-h/d	25.53 (15.17)	25.35 (15.70)	26.83 (15.00)	21.95 (13.44)	<0.001
Smoking					
Non-smoker	32,378 (60.78%)	15,019 (68.36%)	12,372 (52.09%)	4987 (66.07%)	<0.001
Occasional smoker	2514 (4.72%)	747 (3.40%)	1320 (5.56%)	447 (5.92%)	
Ever-smoker	3005 (5.64%)	997 (4.54%)	1511 (6.36%)	497 (6.58%)	
Current smokers	15,372 (28.86%)	5207 (23.70%)	8548 (35.99%)	1617 (21.42%)	
Alcohol consumption					
Non-drinker	31,323 (58.80%)	15,228 (69.36%)	12,027 (50.64%)	4068 (53.90%)	<0.001
Ever-drinker	2607 (4.89%)	901 (4.10%)	1293 (5.44%)	413 (5.47%)	
Occasional drinker	10,048 (18.86%)	3018 (13.74%)	4997 (21.04%)	2033 (26.93%)	
Current drinker	9291 (17.44%)	2823 (12.85%)	5434 (22.88%)	1034 (13.70%)	
Body mass index, kg/m^2^	24.03 (3.21)	23.88 (3.24)	24.23 (3.18)	23.84 (3.15)	<0.001
Systolic blood pressure, mmHg	132.76 (20.32)	134.45 (21.32)	132.34 (19.70)	129.10 (18.68)	<0.001
Menopausal status					
Non-menopausal	12,487 (40.41%)	5048 (34.71%)	5203 (44.59%)	2236 (47.69%)	<0.001
Undergoing menopause	1900 (6.15%)	932 (6.41%)	747 (6.40%)	221 (4.71%)	
Post-menopausal	16,514 (53.44%)	8563 (58.88%)	5719 (49.01%)	2232 (47.60%)	
Baseline diabetes	2442 (4.58%)	892 (4.06%)	1035 (4.36%)	515 (6.82%)	<0.001
Baseline hypertension	21,129 (39.66%)	9163 (41.71%)	9306 (39.18%)	2660 (35.24%)	<0.001
Family history of stroke	10,310 (19.78%)	4078 (19.01%)	4626 (19.86%)	1606 (21.78%)	<0.001
Family history of myocardial infarction	1303 (2.51%)	443 (2.07%)	587 (2.53%)	273 (3.71%)	<0.001
Family history of diabetes	3508 (6.74%)	1137 (5.31%)	1580 (6.80%)	791 (10.74%)	<0.001
Family history of cancer	13,392 (25.66%)	5480 (25.54%)	5992 (25.68%)	1920 (25.98%)	0.755

Note: Cluster 1: Traditional/Preserved-Heavy cluster; cluster 2: Meat-Centric cluster; cluster 3: Plant-and-Dairy-Abundant cluster.

**Table 3:** Associations between dietary clusters and risk of all-cause and cause-specific mortality.

	Events/Participants	Mortality Rate (Per 1000 Person-Years)	Hazard Ratio (95% CI)					
			Model 1			Model 2		
			Traditional/Preserved-Heavy	Meat-Centric	Plant-and-Dairy-Abundant	Traditional/Preserved-Heavy	Meat-Centric	Plant-and-Dairy-Abundant
Healthy adults								
All-cause mortality	1263/29,605	3.57	1.05 (0.94–1.19)	1	0.83 (0.68–1.00)	0.97 (0.86–1.10)	1	0.93 (0.76–1.13)
CVD mortality	189/29,605	0.53	1.22 (0.89–1.68)	1	0.98 (0.60–1.60)	1.09 (0.79–1.50)	1	1.12 (0.67–1.89)
Diabetes-related mortality	6/29,605	0.02	0.93 (0.18–4.80)	1	——	0.93 (0.19–4.61)	1	——
Cancer mortality	740/29,605	2.09	1.01 (0.86–1.18)	1	0.92 (0.73–1.17)	0.95 (0.81–1.11)	1	1.05 (0.82–1.35)
Patients with T2D								
All-cause mortality	351/2442	12.69	1.25 (0.98–1.58)	1	0.61 (0.45–0.83)	1.12 (0.88–1.42)	1	0.61 (0.44–0.85)
CVD mortality	98/2442	3.54	1.58 (1.01–2.47)	1	0.50 (0.27–0.93)	1.48 (0.93–2.34)	1	0.47 (0.24–0.91)
Diabetes-related mortality	59/2442	2.13	1.08 (0.62–1.87)	1	0.24 (0.09–0.64)	0.82 (0.47–1.45)	1	0.25 (0.09–0.71)
Cancer mortality	122/2442	4.41	0.90 (0.60–1.36)	1	0.72 (0.45–1.16)	0.87 (0.57–1.33)	1	0.71 (0.43–1.18)
Patients with hypertension								
All-cause mortality	2410/21,129	9.76	1.20 (1.10–1.31)	1	0.87 (0.76–1.00)	1.13 (1.03–1.23)	1	0.93 (0.81–1.08)
CVD mortality	829/21,129	3.36	1.28 (1.10–1.49)	1	0.89 (0.70–1.12)	1.17 (1.00–1.37)	1	0.96 (0.75–1.24)
Diabetes-related mortality	66/21,129	0.27	1.10 (0.65–1.85)	1	0.57 (0.22–1.49)	1.10 (0.68–1.78)	1	0.40 (0.16–0.98)
Cancer mortality	1025/21,129	4.15	1.01 (0.89–1.16)	1	0.85 (0.69–1.04)	0.99 (0.86–1.13)	1	0.92 (0.74–1.15)

Note: Cluster 1: Traditional/Preserved-Heavy cluster; cluster 2: Meat-Centric cluster; cluster 3: Plant-and-Dairy-Abundant cluster. Model 1: adjusted for age and sex. Model 2: further adjusted for the following variables on the basis of Model 1: highest education level (primary school or below, middle school/technical secondary school, college or above), household annual income (<20,000, 20,000–34,999, ≥35,000 yuan/year), marital status (married, widowed/separated/divorced, unmarried), physical activity level (metabolic equivalent of task hours per day, MET-h/d), smoking (non-smoker: lifetime smoking ≤100 cigarettes; occasional smoker; ever-smoker: cessation for at least 6 months; current smokers: <10 cigarettes/day and ≥10 cigarettes/day), alcohol consumption (non-drinker: never or almost never; ever-drinker: currently does not drink but previously drank at least weekly for ≥1 year; occasional drinker: drinking only on special occasions, seasons, or less than once per week; current drinker: drinking at least once per week), body mass index (BMI, kg/m^2^), and menopausal status for women (not yet menopausal, currently undergoing menopause, post-menopausal). Additionally, family history of specific diseases was adjusted for, stratified by cause of death as follows: for all-cause mortality, adjusted for family history of stroke, myocardial infarction, diabetes, and malignant cancer (yes/no); for cardiovascular mortality, adjusted for family history of stroke and myocardial infarction (yes/no); for diabetes-related mortality, adjusted for family history of diabetes (yes/no); for malignant cancer mortality, adjusted for family history of malignant cancer (yes/no). For participants with T2D, the model was additionally adjusted for diabetes duration (years) and history of hypertension (yes/no); for participants with hypertension, the model was additionally adjusted for baseline history of diabetes (yes/no) and mean systolic blood pressure (mmHg).

## Data Availability

The data that support the findings of this study are available from the Department of the China Kadoorie Biobank, but restrictions apply to the availability of these data, which were used under license for the current study and are not publicly available. Data are, however, available from the authors upon reasonable request and with the permission of the Department of the China Kadoorie Biobank.

## Supplementary Materials

The following supporting information can be downloaded at: https://www.mdpi.com/article/10.3390/nu18111816/s1, Figure S1. Elbow plot of within-cluster sum of squares (WSS) for the K-means clustering analysis. Figure S2. Radar plot of standardized dietary clusters under the alternative k = 2 clustering solution. Figure S3. Radar plot of standardized dietary clusters under the alternative k = 4 clustering solution. Figure S4. Distribution of adjusted Rand index (ARI) values across repeated K-means clustering solutions with k fixed at 3. Figure S5. Subgroup analysis of the associations between dietary clusters and all-cause mortality risk among healthy adults. Figure S6. Subgroup analysis of the associations between dietary clusters and all-cause mortality risk among patients with hypertension. Table S1. Characteristics of baseline healthy population according to dietary cluster. Table S2. Baseline characteristics of participants with T2D according to dietary cluster. Table S3. Baseline characteristics of participants with hypertension according to dietary cluster. Table S4. Sensitivity analysis for the associations between dietary clusters and mortality risk [HR (95% CI)].

## Abbreviations

The following abbreviations are used in this manuscript:

T2D	Type 2 Diabetes
CKB	China Kadoorie Biobank
HRs	Hazard Ratios
CIs	Confidence Intervals
CVD	Cardiovascular Disease
AHEI	Alternative Healthy Eating Index
DASH	Dietary Approaches to Stop Hypertension
FFQ	Food Frequency Questionnaire
WSS	Within-Cluster Sum of Squares
ARI	Adjusted Rand Index
BMI	Body Mass Index
ICD-10	International Classification of Diseases, 10th Revision
SDs	Standard Deviations
CRP	C-Reactive Protein
TNF-α	Tumor Necrosis Factor-α
IL-6	Interleukin-6
NHANES	National Health and Nutrition Examination Survey
LDL-C	Low-Density Lipoprotein Cholesterol
HDL-C	High-Density Lipoprotein Cholesterol
non-HDL-C	Non-High-Density Lipoprotein Cholesterol
ApoB	Apolipoprotein B
Lp(a)	Lipoprotein(a)
