# Supplementary material for: Dietary Clusters and Mortality Risk in a Chinese Population: The Role of Type 2 Diabetes and Hypertension
Source: Nutrients. 2026 Jun 4;18(11):1816. doi: 10.3390/nu18111816 (PMC13258870; doi:10.3390/nu18111816)
Supplement: Supplementary file 1 [file nutrients-18-01816-s001.zip › nutrients-4335470-supplementary.pdf]

Additional File 1

List of contents:

Supplementary Figure S1. Elbow plot of within-cluster sum of squares (WSS) for the K-means clustering analysis.

Supplementary Figure S2. Radar plot of standardized dietary clusters under the alternative k=2 clustering solution.

Supplementary Figure S3. Radar plot of standardized dietary clusters under the alternative k=4 clustering solution.

Supplementary Figure S4. Distribution of adjusted Rand index (ARI) values across repeated K-means clustering solutions with k fixed at 3.

Supplementary Table S1. Characteristics of baseline healthy population according to dietary cluster.

Supplementary Table S2. Baseline characteristics of participants with T2D according to dietary cluster.

Supplementary Table S3. Baseline characteristics of participants with hypertension according to dietary cluster.

Supplementary Figure S5. Subgroup analysis of the associations between dietary clusters and all-cause mortality risk among healthy adults.

Supplementary Figure S6. Subgroup analysis of the associations between dietary clusters and all-cause mortality risk among patients with hypertension.

Supplementary Table S4. Sensitivity analysis for the associations between dietary clusters and mortality risk [HR (95% CI)].

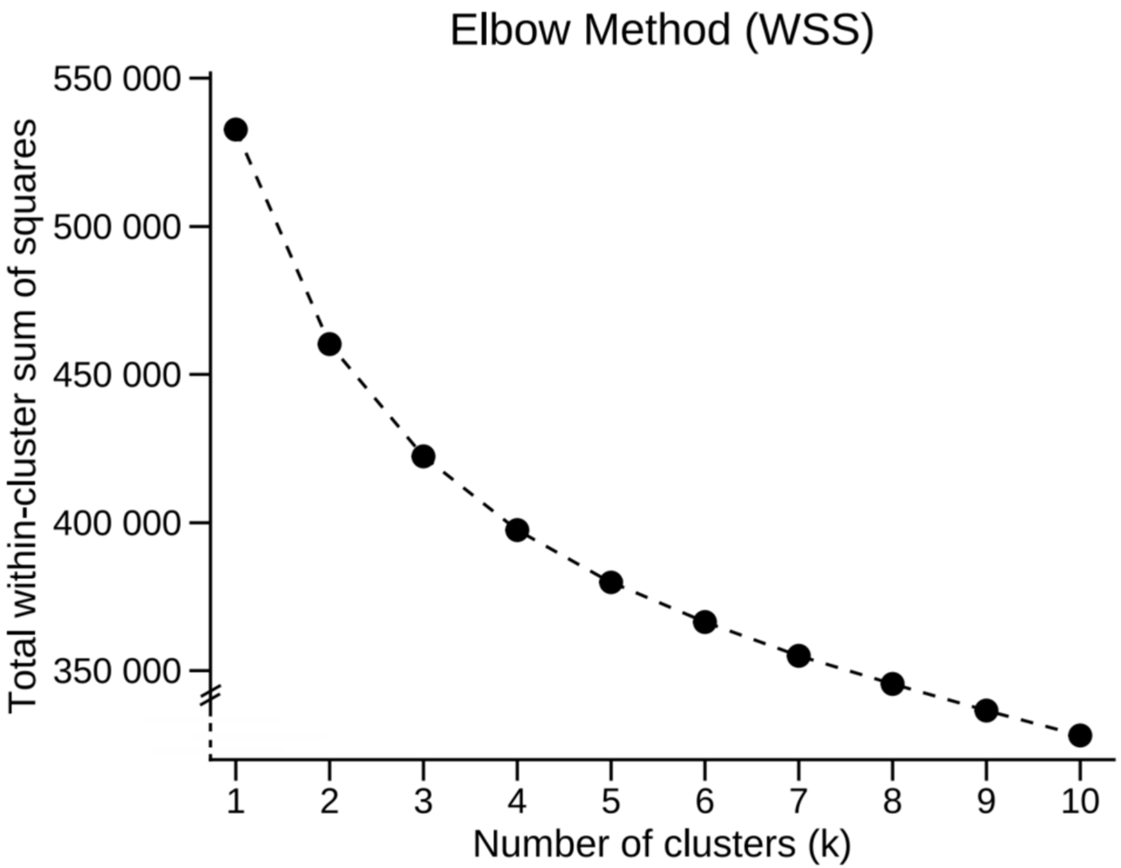

Supplementary Figure S1. Elbow plot of within-cluster sum of squares (WSS) for the K-means clustering analysis. The plot shows the total WSS for candidate cluster solutions with *k* values ranging from 1 to 10. The reduction in WSS was substantial from *k* = 1 to *k* = 3, and became more gradual thereafter, supporting the selection of three clusters.

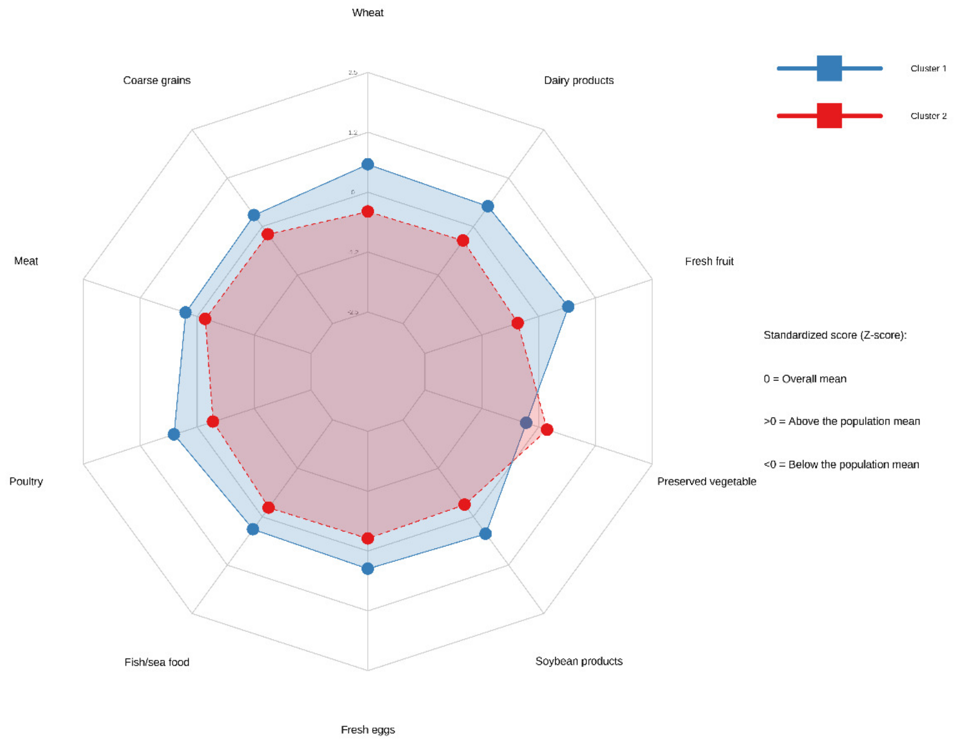

***Supplementary Figure S2*.** Radar plot of standardized dietary clusters under the alternative *k*=2 clustering solution. The plot shows the mean standardized scores (Z-scores) of the 10 food groups included in the cluster analysis for each dietary cluster. Dietary clusters were identified using K-means clustering based on standardized dietary variables. Higher values indicate intake frequencies above the population mean, whereas lower values indicate intake frequencies below the population mean. Under the *k*=2 solution, cluster 1 was characterized by generally higher intake frequencies across most food groups, particularly wheat, coarse grains, poultry, fish/seafood, eggs, soybean products, fresh fruit, and dairy products, with relatively lower preserved vegetable intake. cluster 2 was characterized by lower overall intake frequencies across most food groups and relatively higher preserved vegetable intake. This two-cluster solution mainly separated a broader higher-intake profile from a lower-diversity, preserved-vegetable-heavy profile, but did not distinguish the meat-centered and plant-and-dairy-abundant profiles observed in the three-cluster solution.

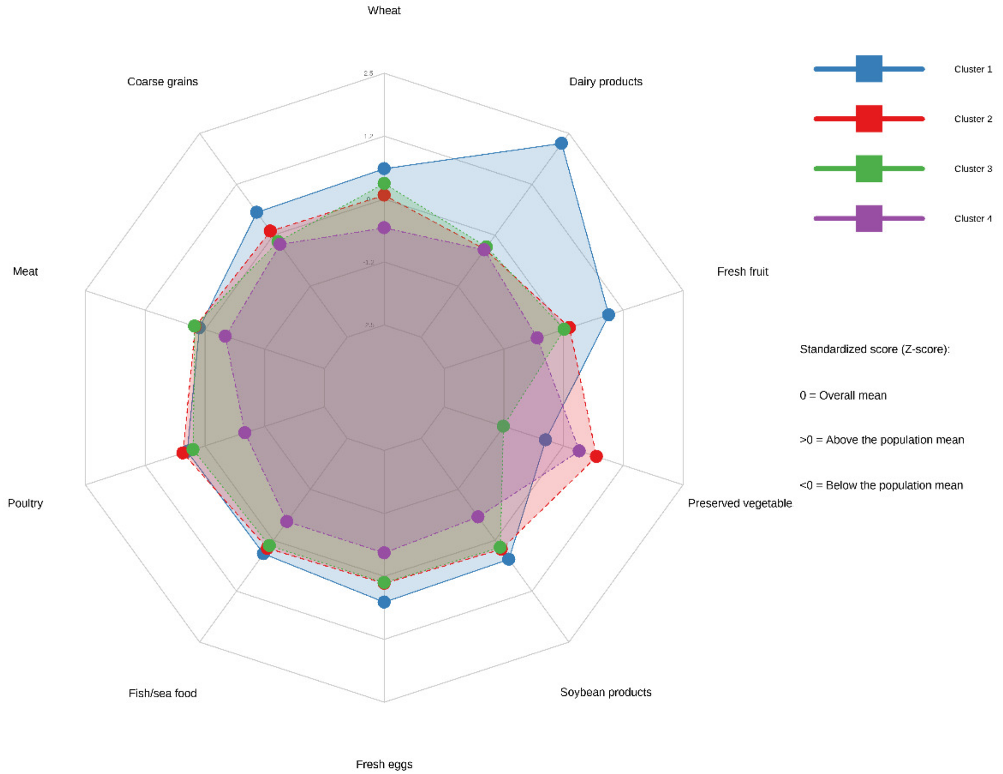

Supplementary Figure S3. Radar plot of standardized dietary clusters under the alternative *k*=4 clustering solution. The plot shows the mean standardized scores (Z-scores) of the 10 food groups included in the cluster analysis for each dietary cluster. Dietary clusters were identified using K-means clustering based on standardized dietary variables. Higher values indicate intake frequencies above the population mean, whereas lower values indicate intake frequencies below the population mean. Under the *k*=4 solution, cluster 1 resembled a fruit, dairy, and soybean product-abundant cluster, while cluster 4 resembled a lower-diversity, preserved-vegetable-heavy cluster. Cluster 2 and cluster 3 were both meat-centered profiles and differed mainly in preserved vegetable intake. Therefore, the four-clusters solution appeared to further subdivide the meat-centered cluster rather than generate an additional clearly distinct dietary cluster.

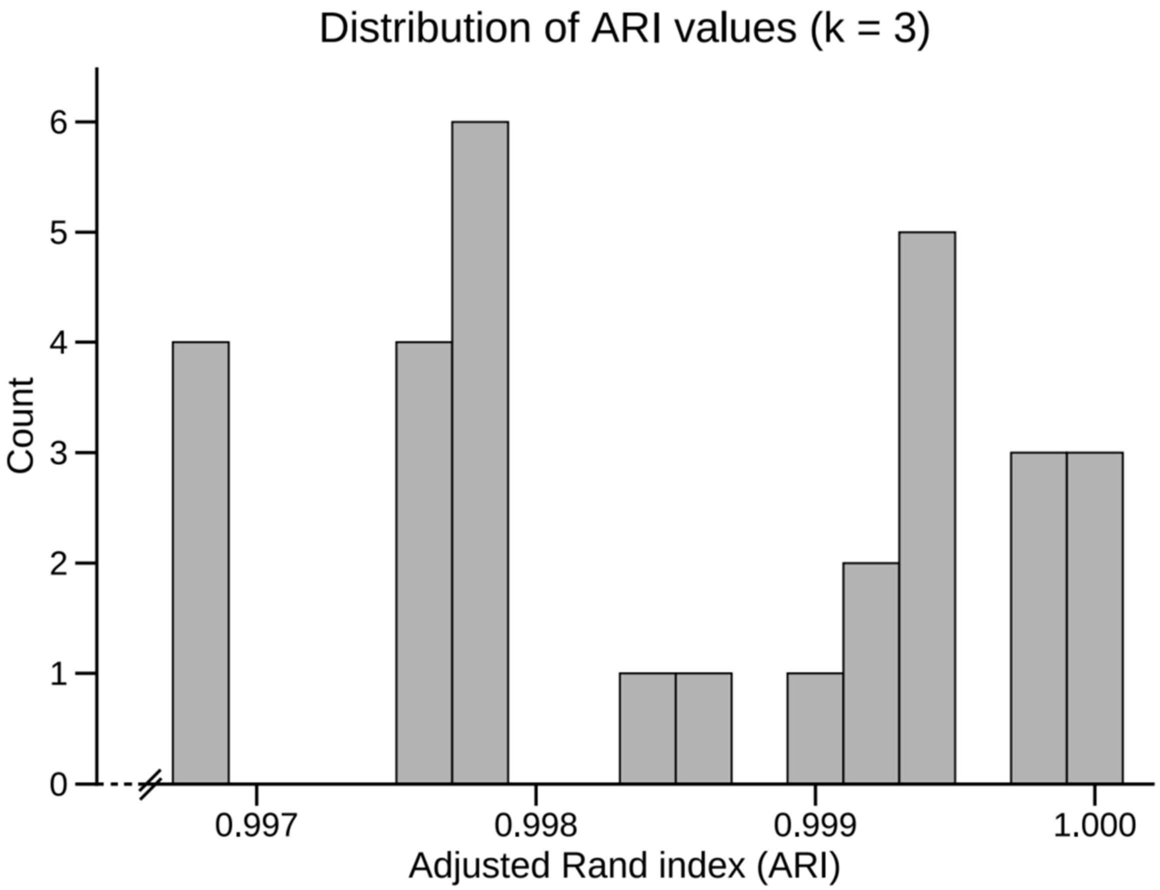

Supplementary Figure S4. Distribution of adjusted Rand index (ARI) values across repeated K-means clustering solutions with *k* fixed at 3. K-means clustering was repeated using different random seeds, and the *ARI* was calculated by comparing each solution with the reference clustering result. The *ARI* values ranged from 0.997 to 1.000, indicating high stability of the clustering results.

Supplementary Table S1. Characteristics of baseline healthy population according to dietary cluster

Characteristics	Traditional/Preserved-Heavy	Meat-Centric	Plant-and-Dairy-Abundant	*P*
No. of participants	11827	13413	4365	
Age, y	50.22 (9.42)	48.07 (9.13)	46.90 (9.76)	<0.001
Women	7901 (66.80%)	6925 (51.63%)	2897 (66.37%)	<0.001
Highest education level				<0.001
primary school or below	4192 (35.44%)	2520 (18.79%)	381 (8.73%)	
middle/technical secondary school	7183 (60.73%)	9458 (70.51%)	2818 (64.56%)	
college or above	452 (3.83%)	1435 (10.70%)	1166 (26.71%)	
Household income				<0.001
<20,000 RMB/year	3752 (31.73%)	2192 (16.34%)	463 (10.60%)	
20,000~34,999 RMB/year	4576 (38.69%)	4300 (32.06%)	1123 (25.73%)	
≥35,000 RMB/year	3499 (29.58%)	6921 (51.60%)	2779 (63.67%)	
Marital status				<0.001
married	11019 (93.17%)	12865 (95.91%)	4119 (94.36%)	
widowed/separated/divorced	767 (6.49%)	528 (3.94%)	233 (5.34%)	
unmarried	41 (0.35%)	20 (0.15%)	13 (0.30%)	
Physical activity level, MET-h/d	27.91 (15.34)	28.61 (14.52)	24.47 (13.37)	<0.001
Smoking				<0.001
non-smoker	8115 (68.61%)	7229 (53.90%)	3008 (68.91%)	
occasional smoker	398 (3.37%)	711 (5.30%)	226 (5.18%)	
ever-smoker	360 (3.04%)	561 (4.18%)	164 (3.76%)	
current smokers	2954 (24.98%)	4912 (36.62%)	967 (22.15%)	
Alcohol consumption				<0.001

non-drinker	8137 (68.80%)	6861 (51.15%)	2321 (53.17%)	
ever-drinker	353 (2.98%)	497 (3.71%)	165 (3.78%)	
occasional drinker	1885 (15.94%)	3151 (23.49%)	1301 (29.81%)	
current drinker	1452 (12.28%)	2904 (21.65%)	578 (13.24%)	
Body mass index, kg/m²	23.23 (2.94)	23.56 (2.94)	23.09 (2.90)	<0.001
Menopausal status				<0.001
non menopause	3726 (47.16%)	3982 (57.51%)	1834 (63.31%)	
undergoing menopause	552 (6.99%)	418 (6.04%)	131 (4.52%)	
postmenopausal	3623 (45.85%)	2524 (36.45%)	932 (32.17%)	
Family history of stroke	1860 (16.03%)	2091 (15.84%)	724 (16.87%)	0.277
Family history of myocardial infarction	234 (2.02%)	287 (2.18%)	126 (2.94%)	0.002
Family history of diabetes	544 (4.69%)	802 (6.08%)	405 (9.44%)	<0.001
Family history of cancer	2968 (25.56%)	3308 (25.02%)	1045 (24.30%)	0.246

Note: Cluster 1: Traditional/Preserved-Heavy cluster; Cluster 2: Meat-Centric cluster; Cluster 3: Plant-and-Dairy-Abundant cluster. The baseline healthy population was defined as participants without coronary heart disease, stroke or transient ischemic attack, cancer, emphysema and chronic bronchitis, T2D, or hypertension at baseline.

Supplementary Table S2. Baseline characteristics of participants with T2D according to dietary cluster

Characteristics	Traditional/Preserved-Heavy	Meat-Centric	Plant-and-Dairy-Abundant	*P*
No. of participants	892	1035	515	
Age, y	57.49 (9.00)	55.80 (9.02)	59.39 (9.05)	<0.001
Women	586 (65.70%)	497 (48.02%)	289 (56.12%)	<0.001
Highest education level				<0.001
primary school or below	453 (50.78%)	290 (28.02%)	86 (16.70%)	
middle/technical secondary school	411 (46.08%)	636 (61.45%)	314 (60.97%)	
college or above	28 (3.14%)	109 (10.53%)	115 (22.33%)	
Household income				<0.001
<20,000 RMB/year	365 (40.92%)	242 (23.38%)	115 (22.33%)	
20,000~34,999 RMB/year	259 (29.04%)	271 (26.18%)	146 (28.35%)	
≥35,000 RMB/year	268 (30.04%)	522 (50.43%)	254 (49.32%)	
Marital status				<0.001
married	792 (88.79%)	970 (93.72%)	474 (92.04%)	
widowed/separated/divorced	95 (10.65%)	63 (6.09%)	41 (7.96%)	
unmarried	5 (0.56%)	2 (0.19%)	0 (0.00%)	
Physical activity level, MET-h/d	19.88 (15.08)	22.08 (15.01)	15.35 (11.21)	<0.001
Smoking				<0.001
non-smoker	616 (69.06%)	534 (51.59%)	311 (60.39%)	
occasional smoker	37 (4.15%)	59 (5.70%)	21 (4.08%)	
ever-smoker	66 (7.40%)	90 (8.70%)	61 (11.84%)	
current smokers	173 (19.4%)	352 (34.01%)	122 (23.69%)	
Alcohol consumption				<0.001

non-drinker	644 (72.20%)	546 (52.75%)	316 (61.36%)	
ever-drinker	63 (7.06%)	93 (8.99%)	47 (9.13%)	
occasional drinker	93 (10.43%)	188 (18.16%)	88 (17.09%)	
current drinker	92 (10.31%)	208 (20.10%)	64 (12.43%)	
Body mass index, kg/m²	25.28 (3.53)	25.37 (3.41)	24.65 (3.30)	<0.001
Menopausal status				<0.001
non menopause	99 (16.89%)	95 (19.11%)	29 (10.03%)	
undergoing menopause	30 (5.12%)	41 (8.25%)	9 (3.11%)	
postmenopausal	457 (78.0%)	361 (72.64%)	251 (86.85%)	
Family history of stroke	199 (22.95%)	244 (24.21%)	136 (27.20%)	0.209
Family history of myocardial infarction	15 (1.73%)	34 (3.38%)	21 (4.20%)	0.02
Family history of diabetes	150 (17.34%)	193 (19.13%)	121 (24.20%)	0.008
Family history of cancer	248 (28.57%)	276 (27.35%)	149 (29.80%)	0.597

Note: Cluster 1: Traditional/Preserved-Heavy cluster; Cluster 2: Meat-Centric cluster; Cluster 3: Plant-and-Dairy-Abundant cluster. Participants were classified as having baseline T2D if they had fasting blood glucose ≥7.0 mmol/L, or random blood glucose ≥11.1 mmol/L, or self-reported or physician-diagnosed diabetes, or current use of any glucose-lowering medications at baseline, with individuals with type 1 diabetes (ICD-10: E10) excluded.

Supplementary Table S3. Baseline characteristics of participants with hypertension according to dietary cluster

Characteristics	Traditional/Preserved-Heavy	Meat-Centric	Plant-and-Dairy-Abundant	*P*
No. of participants	9163	9306	2660	
Age, y	57.88 (9.67)	55.14 (9.68)	57.23 (10.36)	<0.001
Women	6000 (65.5%)	4241 (45.6%)	1484 (55.8%)	<0.001
Highest education level				<0.001
primary school or below	4913 (53.6%)	2794 (30.0%)	423 (15.9%)	
middle/technical secondary school	3984 (43.5%)	5659 (60.8%)	1489 (56.0%)	
college or above	266 (2.9%)	853 (9.2%)	748 (28.1%)	
Household income				<0.001
<20,000 RMB/year	4229 (46.2%)	2328 (25.0%)	487 (18.3%)	
20,000~34,999 RMB/year	2632 (28.7%)	2678 (28.8%)	714 (26.8%)	
≥35,000 RMB/year	2302 (25.1%)	4300 (46.2%)	1459 (54.8%)	
Marital status				<0.001
married	7952 (86.8%)	8658 (93.0%)	2434 (91.5%)	
widowed/separated/divorced	1162 (12.7%)	628 (6.7%)	223 (8.4%)	
unmarried	49 (0.5%)	20 (0.2%)	3 (0.1%)	
Physical activity level, MET-h/d	22.24 (15.49)	24.53 (15.28)	18.50 (12.69)	<0.001
Smoking				<0.001
non-smoker	6238 (68.1%)	4601 (49.4%)	1652 (62.1%)	
occasional smoker	316 (3.4%)	547 (5.9%)	188 (7.1%)	
ever-smoker	563 (6.1%)	830 (8.9%)	267 (10.0%)	
current smokers	2046 (22.3%)	3328 (35.8%)	553 (20.8%)	
Alcohol consumption				<0.001
non-drinker	6397 (69.8%)	4635 (49.8%)	1443 (54.2%)	

ever-drinker	484 (5.3%)	685 (7.4%)	214 (8.0%)	
occasional drinker	1002 (10.9%)	1635 (17.6%)	604 (22.7%)	
current drinker	1280 (14.0%)	2351 (25.3%)	399 (15.0%)	
Body mass index, kg/m²	24.75 (3.39)	25.24 (3.24)	25.14 (3.15)	<0.001
Systolic blood pressure, mmHg	152.79 (18.84)	149.87 (17.69)	146.65 (17.29)	<0.001
Menopausal status				<0.001
non menopause	1107 (18.4%)	1026 (24.2%)	310 (20.9%)	
undergoing menopause	334 (5.6%)	287 (6.8%)	73 (4.9%)	
postmenopausal	4559 (76.0%)	2928 (69.0%)	1100 (74.2%)	
Family history of stroke	2053 (23.1%)	2356 (25.9%)	761 (29.5%)	<0.001
Family history of myocardial infarction	189 (2.1%)	273 (3.0%)	119 (4.6%)	<0.001
Family history of diabetes	498 (5.6%)	668 (7.4%)	293 (11.4%)	<0.001
Family history of cancer	2242 (25.2%)	2382 (26.2%)	727 (28.2%)	0.011

Note: Cluster 1: Traditional/Preserved-Heavy cluster; Cluster 2: Meat-Centric cluster; Cluster 3: Plant-and-Dairy-Abundant cluster. Patients with hypertension were defined as those with systolic blood pressure ≥140 mmHg, or diastolic blood pressure ≥90 mmHg, or self-reported or physician-diagnosed hypertension, or current use of antihypertensive medications.

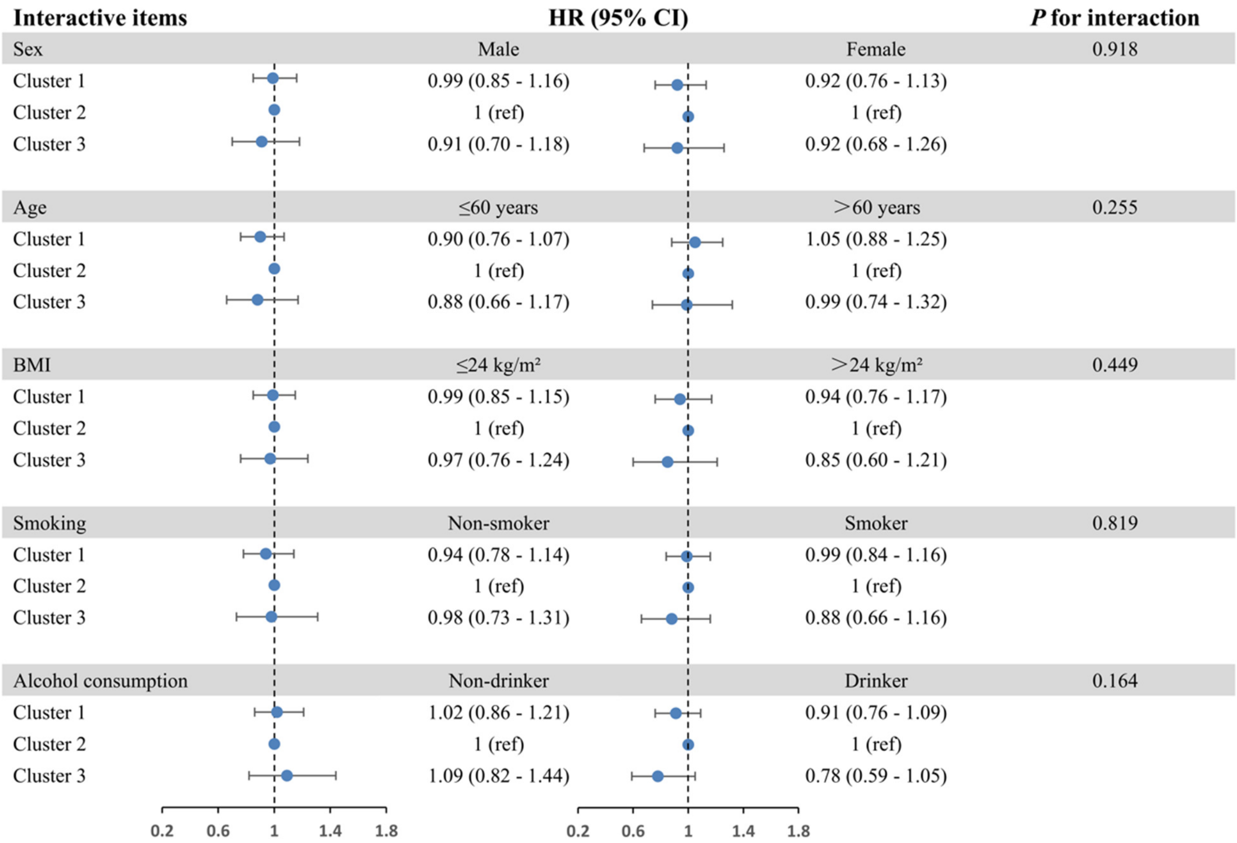

Supplementary Figure S5. Subgroup analysis of the associations between dietary clusters and all-cause mortality risk among healthy adults. Cluster 1: Traditional/Preserved-Heavy cluster; Cluster 2: Meat-Centric cluster; Cluster 3: Plant-and-Dairy-Abundant cluster. The HRs (95% CIs) for all-cause mortality were estimated using Cox proportional hazards models, with cluster 2 as the reference group. Analyses were stratified by sex, age, body mass index (BMI), smoking status, and alcohol consumption. The fully adjusted model included age, sex, highest education level, annual household income, marital status, physical activity, smoking, alcohol consumption, BMI, menopausal status in women, and family history of relevant diseases. For all-cause mortality, family history of stroke, myocardial infarction, diabetes, and cancer was additionally adjusted for. BMI: body mass index; CIs: confidence intervals; HRs: hazard ratios.

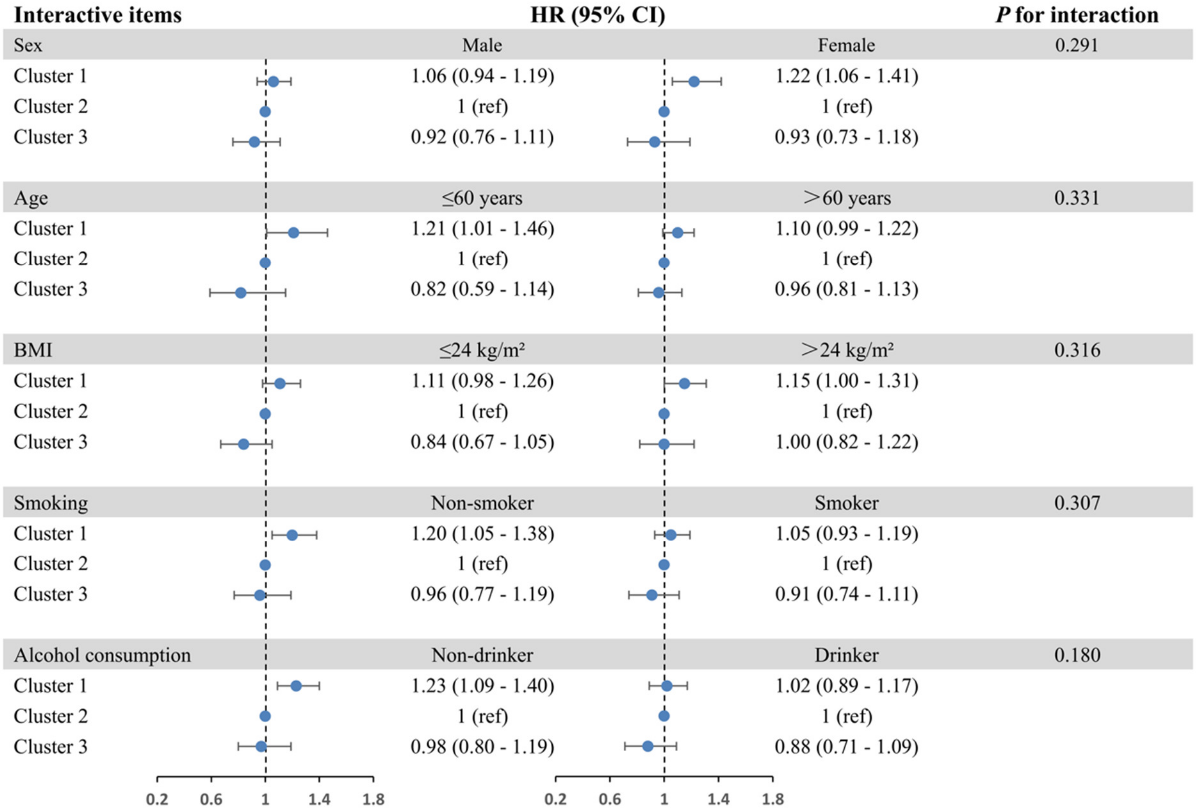

Interactive items	HR (95% CI)		*P* for interaction
**Sex**	Male	Female	0.291
Cluster 1	1.06 (0.94 - 1.19)	1.22 (1.06 - 1.41)	
Cluster 2	1 (ref)	1 (ref)	
Cluster 3	0.92 (0.76 - 1.11)	0.93 (0.73 - 1.18)	
**Age**	≤60 years	>60 years	0.331
Cluster 1	1.21 (1.01 - 1.46)	1.10 (0.99 - 1.22)	
Cluster 2	1 (ref)	1 (ref)	
Cluster 3	0.82 (0.59 - 1.14)	0.96 (0.81 - 1.13)	
**BMI**	≤24 kg/m²	>24 kg/m²	0.316
Cluster 1	1.11 (0.98 - 1.26)	1.15 (1.00 - 1.31)	
Cluster 2	1 (ref)	1 (ref)	
Cluster 3	0.84 (0.67 - 1.05)	1.00 (0.82 - 1.22)	
**Smoking**	Non-smoker	Smoker	0.307
Cluster 1	1.20 (1.05 - 1.38)	1.05 (0.93 - 1.19)	
Cluster 2	1 (ref)	1 (ref)	
Cluster 3	0.96 (0.77 - 1.19)	0.91 (0.74 - 1.11)	
**Alcohol consumption**	Non-drinker	Drinker	0.180
Cluster 1	1.23 (1.09 - 1.40)	1.02 (0.89 - 1.17)	
Cluster 2	1 (ref)	1 (ref)	
Cluster 3	0.98 (0.80 - 1.19)	0.88 (0.71 - 1.09)	

Supplementary Figure S6. Subgroup analysis of the associations between dietary clusters and all-cause mortality risk among patients with hypertension. Cluster 1: Traditional/Preserved-Heavy cluster; Cluster 2: Meat-Centric cluster; Cluster 3: Plant-and-Dairy-Abundant cluster. The HRs (95% CIs) for all-cause mortality were estimated using Cox proportional hazards models, with cluster 2 as the reference group. Analyses were stratified by sex, age, body mass index (BMI), smoking status, and alcohol consumption. The fully adjusted model included age, sex, highest education level, annual household income, marital status, physical activity, smoking, alcohol consumption, BMI, menopausal status in women, family history of relevant diseases, baseline diabetes status, and systolic blood pressure. For all-cause mortality, family history of stroke, myocardial infarction, diabetes, and cancer was additionally adjusted for. BMI: body mass index; CIs: confidence intervals; HRs: hazard ratios.

Supplementary Table S4. Sensitivity analysis for the associations of dietary clusters with mortality risk [HR (95% CI)]

	Healthy adults				Patients with T2D				Patients with hypertension			
	Events/N	Traditional/Preserved-Heavy	Meat-Centric	Plant-and-Dairy-Abundant	Events/N	Traditional/Preserved-Heavy	Meat-Centric	Plant-and-Dairy-Abundant	Events/N	Traditional/Preserved-Heavy	Meat-Centric	Plant-and-Dairy-Abundant
Participants who died during the first year of follow-up were excluded												
All-cause	1240/29900	0.96 (0.85-1.09)	1	0.90 (0.74-1.10)	338/2429	1.11 (0.86-1.42)	1	0.59 (0.42-0.81)	2330/21049	1.13 (1.03-1.24)	1	0.93 (0.80-1.08)
CVD	191/29900	1.04 (0.76-1.44)	1	1.00 (0.59-1.69)	96/2429	1.41 (0.89-2.24)	1	0.46 (0.24-0.90)	801/21049	1.17 (1.00-1.37)	1	0.97 (0.75-1.25)
Diabetes	9/29900	0.73 (0.18-2.91)	1	1.17 (0.15-9.38)	57/2429	0.76 (0.43-1.36)	1	0.26 (0.09-0.73)	64/21049	1.05 (0.64-1.72)	1	0.40 (0.16-0.99)
Cancer	722/29900	0.95 (0.80-1.11)	1	1.03 (0.80-1.32)	116/2429	0.93 (0.60-1.43)	1	0.68 (0.40-1.13)	989/21049	0.99 (0.86-1.14)	1	0.89 (0.71-1.11)

Note: Cluster 1: Traditional/Preserved-Heavy cluster; Cluster 2: Meat-Centric cluster; Cluster 3: Plant-and-Dairy-Abundant cluster. The HRs (95% CIs) for these sensitivity analyses were estimated using Cox proportional hazards models after excluding participants who died within the first year of follow-up, with adjustment for the same covariates as Model 2 in the main analysis.
